# Diffusion Tensor Imaging of the Auditory Neural Pathway for Clinical Outcome of Cochlear Implantation in Pediatric Congenital Sensorineural Hearing Loss Patients

**DOI:** 10.1371/journal.pone.0140643

**Published:** 2015-10-20

**Authors:** Lexing Huang, Wenbin Zheng, Chunxiao Wu, Xiaoqin Wei, Xianguang Wu, Yanting Wang, Hongyi Zheng

**Affiliations:** 1 Department of Radiology, The Second Affiliated Hospital, Medical College of Shantou University, Shantou, China; 2 Department of E.N.T, The Second Affiliated Hospital, Medical College of Shantou University, Shantou, China; Duke-NUS Graduate Medical School, SINGAPORE

## Abstract

Although conventional structural MRI provides vital information in the evaluation of congenital sensorineural hearing loss (SNHL), it is relatively insensitive to white matter microstructure. Our objective was to evaluate possible changes in microstructure of the auditory pathway in children with congenital sensorineural hearing loss (SNHL), and the possible distinction between good and poor outcome of cochlear implantation (CI) patients by using diffusion tensor imaging (DTI). Twenty-four patients with congenital SNHL and 20 healthy controls underwent conventional MRI and DTI examination using a 1.5T MR scanner. The DTI metrics of fractional anisotropy (FA) and mean diffusivity (MD) of six regions of interest (ROIs) positioned along the auditory pathway—the trapezoid body, superior olivary nucleus, inferior colliculus, medial geniculate body, auditory radiation and white matter of Heschl's gyrus—was measured in all subjects. Among the 24 patients, 8 patients with a categorie of auditory performance (CAP) score over 6 were classified into the good outcome group, and 16 patients with a CAP score below 6 were classified into the poor outcome group. A significant decrease was observed in FA values while MD values remained unchanged at the six ROIs of SNHL patients compared with healthy controls. Compared to good outcome subjects, poor outcome subjects displayed decreased FA values at all of the ROIs. No changes were observed in MD values. Correlation analyses only revealed strong correlations between FA values and CAP scores, and strong correlations between CAP scores and age at implant were also found. No correlations of FA values with age at implant were observed. Our results show that preoperative DTI can be used to evaluate microstructural alterations in the auditory pathway that are not detectable by conventional MR imaging, and may play an important role in evaluating the outcome of CI. Early cochlear implantation might be more effectively to restore hearing in SNHL patients.

## Introduction

Imaging modalities, such as CT and MRI have been widely used for the evaluation of congenital sensorineural hearing loss. Preoperative high-resolution CT offers the advantage of visualizing any coexistent middle or external ear anomalies and important anatomic variants, and MR imaging provides definitive information about the integrity of the cochlear nerve and the fluid-filled spaces of the inner ear [1]. In addition, preoperative magnetic resonance imaging (MRI) can yield valuable information regarding the status of the inner ear in pediatric cochlear implant (CI) recipients, which may be related to postoperative outcomes [2,3]. However, although these complementary modalities can accurately and objectively evaluate sensorineural hearing loss due to morphological abnormalities present [4], the functional change or dysfunction of the central auditory pathway itself cannot be evaluated by conventional imaging [5]. Such changes or dysfunction of the central auditory pathway may potentially be associated with clinical outcome and rehabilitation in CI patients [6]. Diffusion tensor imaging (DTI) is a sensitive, noninvasive tool for assessing white matter abnormalities in the central nervous system [7]. It detects the orientation of water molecules and yields an index of microstructural integrity through quantification of the directionality of water diffusion [5]. The DTI index of fractional anisotropy (FA) is the normal standard deviation of the diffusivities and represents the degree of anisotropy of water molecules in white matter [8]. Mean diffusivity (MD) provides the overall magnitude of water diffusion [7]. Any factors that lead to changes of tissue density, damage to the myelin sheath and white matter fiber will affect these indices through changing of the directionality of water diffusion. Neural reorganization occurs when the inputs to the sensory system change. Similarly, the pattern of auditory cortical activation becomes altered when the inputs to the auditory system change as a result of peripheral hearing loss. Therefore, we investigated whether microstructural and functional changes of the central auditory pathway could be detected in SNHL patients by using DTI, and investigated the correlations between the clinical outcomes and indices of DTI, and correlations of the clinical outcomes and indices of DTI with age at implant.

## Materials and Methods

### Ethics statement

The study was approved by the ethics committee of The Second Affiliated Hospital of Medical College of Shantou University. All clinical investigations were conducted according to the guidelines of the Declaration of Helsinki. Written informed consent for the study was obtained from the children's legal guardians and included in their hospital medical record.

### Subjects

Twenty-four patients (14 males, 10 females; mean age, 4.7 ± 1.0 years-old) with bilaterally profound sensorineural hearing loss, and 20 healthy controls (10 males, 10 females; mean age, 4.2 ± 2.9 years-old) were recruited for this study. Inclusion criteria were as follows: (1) no morphologic abnormalities of the inner ear, auditory nerve, and central auditory pathway (conventional MRI was acquired before DTI examination). (2) no previous history of otological surgery or systemic ototoxic drug therapy. All the patients were deaf pre-lingually. Hearing level was measured in a sound proof booth using a calibrated pure tone audiometer (GSI 10, USA). The degree of hearing loss was profound, and all patients had bilateral SNHL greater than 90 dB HL (averaged threshold of 500 Hz, 1 and 2 kHz; pure tone average, PTA). Conventional MRI and DTI scans were performed before CI. Among the 24 patients, 8 patients (mean age, 4.6 ± 1.0 years) with a categories of auditory performance (CAP) score over 6 were classified into the good outcome group and the other 16 patients (mean age, 4.7 ± 1.0 years) with a CAP score below 6 were classified into the poor outcome group. There was no difference in age or age at time of CI between two groups (*P*>0.05), and there were significant differences in CI outcome (CAP score) between the good/poor outcome groups (*P*<0.05). The CAP scores of all the patients were administered by a qualified audiologist in accordance with standard protocols as described in the test manuals 12 months post-operation. The audiologist sets the minimum and maximum current level outputs for each electrode in the array based on the user's reports of loudness. The audiologist also selects the appropriate speech processing strategy and program parameters for the user after CI. All the CI patients wore their processors as long as possible according to the audiologist's guidance and tried to use oral communication as much as possible instead of sign communication in their daily life.

Clinical and audiogram data of SNHL patients are summarized in Table 1.

**Table 1:** Clinical and audiogram data of patients.

	Good outcome (n = 8)	Poor outcome (n = 16)	*P*-value
Age	4.6 ± 1.0	4.7 ± 1.0	>0.05
CI (side)	Right (7),left (1)	Right (9),left (7)	-
Age at time of CI	4.7± 1.0	5.2 ±1.0	>0.05
ABR (pre CI)	No response	No response	-
CAP score	6.1 ± 0.35	4.5 ± 0.52	<0.05*

Values are presented as mean±SD.

*Statistically significant difference.

CI, cochlear implantation; ABR, auditory brainstem response; CAP, categories of auditory performance.

### MRI, DTI Acquisition

Conventional MRI and DTI scans were performed using a 1.5T MR imaging system (Signa; GE Healthcare, Milwaukee, Wisconsin) with an eight-channel head coil. We performed conventional MRI before DTI examination to exclude morphologic abnormalities of the inner ear, auditory nerve, and central auditory pathway. Conventional MRI protocols included: (1) T1-flair; (2) T2-weighted fast spin echo; (3) T2-flair; (4) DWI. After conventional MRI acquisition, DTI data was acquired using a single-shot, spin-echo, echo-planar imaging sequence with the parameters as follows: TR, 8000 ms; TE, 99.3 ms; NEX = 1; section thickness, 2 mm; section gap, 0 mm; matrix = 128×128; FOV = 240×240 mm; diffusion tensor, 15 directions; minimum b-value of 0 s/mm2; maximum b-value of 1000 s/mm2.

### Data Analysis

**Fig 1:**
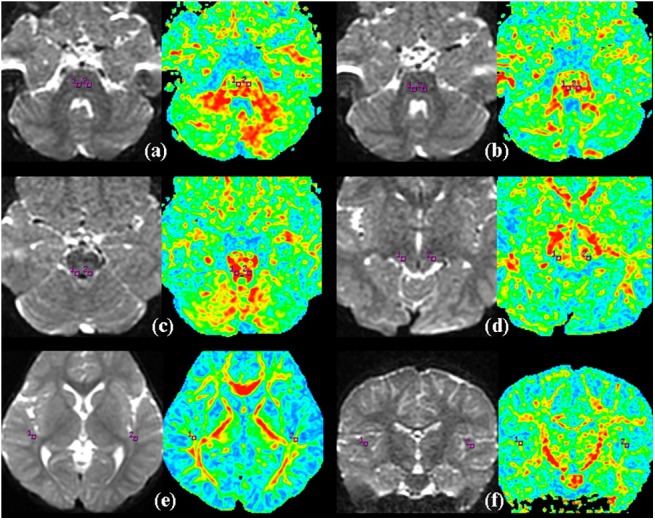
Representative DT imaging of the ROIs. (a) the trapezoid body, (b) superior olivary nucleus, (c) inferior colliculus, (d) medial geniculate body, (e) the auditory radiation, (f) the white matter of Heschl's gyrus, (square box) the selected ROI.

All diffusion tensor analysis and image processing were carried out on an Advantage Workstation for Windows (AW4.5, GE Healthcare). The final DTI dataset was fed into Functool software. After correction for movement and EPI-induced distortion artifacts, the FA and MD maps were automatically computed. Then, FA and MD values at the trapezoid body (TB), superior olivary nucleus (SON), inferior colliculus (IC), medial geniculate body (MGB), auditory radiation (AR) and white matter of Heschl's gyrus (WHG) were measured using a region of interest (ROI) analysis. The ROI size was 14 mm^2^ and was traced on each of the anatomical locations on both sides, as shown in Fig 1.

### Statistical Analysis

ROIs along the central auditory pathway were drawn bilaterally for evaluation. The means and standard deviations of all indices were computed from each region of interest for all subjects. For comparisons within the SNHL group and control group, the Student’s t-test was used with a two-tailed *P*-value<0.05 considered statistically significant. We used Student’s t-test to analyze all indices between good and poor outcome subjects with a statistical threshold *P*-value<0.05. Spearman rank correlation analysis was used to determine correlations between individual indices of DTI and CAP scores. The correlations of the CAP scores and indices of DTI with age at implant were also determined, with a two-tailed *P*-value<0.05 as statistically significant. SPSS ver. 16 (SPSS, Chicago, IL, USA) was used for all statistical analyses.

## Results

### Conventional MRI

Conventional MRI showed no anatomical abnormalities of the brain or inner ear, and no cochlear nerve deficiency in any individual.

### DTI

Student’s t-test evaluation showed that FA values were decreased (*P*<0.05) while MD values remained unchanged at the six ROIs (trapezoid body, superior olivary nucleus, inferior colliculus, medial geniculate body, auditory radiation and white matter of Heschl's gyrus) positioned along the auditory neural pathway in the SNHL group (n = 24) compared to the controls (n = 20) (Tables 2 and 3). Poor outcome subjects displayed decreased FA values at all of the six ROIs and no changes were observed in MD values (Tables 4 and 5).

**Table 2:** Summary of FA values at the TB, SON, IC, MGB, AR and WHG in the SNHL and control groups.

	SNHL (n = 24)	Control (n = 20)	*P*-value
TB	0.37 ± 0.04	0.39 ± 0.02	0.011*
SON	0.36 ± 0.04	0.38 ± 0.01	0.007*
IC	0.44 ± 0.04	0.46 ± 0.02	0.007*
MGB	0.34 ± 0.04	0.36 ± 0.01	0.008*
AR	0.31 ± 0.05	0.38 ± 0.03	0.006*
WHG	0.33 ± 0.04	0.35 ± 0.02	0.002*

Values are presented as mean ± SD

*Statistically significant difference.

FA values measured at the TB, SON, IC, MGB, AR and WHG of the SNHL group were compared with the control group.

**Table 3:** Summary of MD values at the TB, SON, IC, MGB, AR and WHG in the SNHL and control groups.

	SNHL (n = 24)	Control (n = 20)	*P*-value
TB	0.93 ± 0.10	0.91 ± 0.10	0.464
SON	0.89 ± 0.12	0.88 ± 0.11	0.578
IC	0.81 ± 0.10	0.78 ± 0.10	0.224
MGB	0.72 ± 0.13	0.72 ± 0.11	0.976
AR	0.80 ± 0.09	0.80 ± 0.10	0.771
WHG	0.62 ± 0.10	0.61 ± 0.11	0.617

Values are presented as mean ± SD; MD values measured at the TB, SON, IC, MGB, AR and WHG of the SNHL group were compared with the control group.

**Table 4:** Summary of FA values at the TB, SON, IC, MGB, AR and WHG of the good/ poor outcome group of SNHL patients.

	Good outcome (n = 8)	Poor outcome (n = 16)	*P*-value
TB	0.39 ± 0.04	0.36 ± 0.03	0.003*
SON	0.40 ± 0.04	0.35 ± 0.03	0.002*
IC	0.47 ± 0.04	0.43 ± 0.03	0.002*
MGB	0.37 ± 0.04	0.33 ± 0.03	0.001*
AR	0.35 ± 0.05	0.30 ± 0.03	0.002*
WHG	0.37 ± 0.04	0.31 ± 0.02	0.004*

Values are presented as mean±SD

*Statistically significant difference.

FA values measured at the TB, SON, IC, MGB, AR and WHG of the good outcome group of SNHL patients were compared with the poor group.

**Table 5:** Summary of MD values at the TB, SON, IC, MGB, AR and WHG of the good/ poor outcome group of SNHL patients.

	Good outcome (n = 8)	Poor outcome (n = 16)	*P*-value
TB	0.92 ± 0.10	0.93 ± 0.10	0.709
SON	0.91 ± 0.08	0.89 ± 0.13	0.418
IC	0.80 ± 0.10	0.82 ± 0.10	0.622
MGB	0.71 ± 0.09	0.73 ± 0.15	0.659
AR	0.76 ± 0.10	0.82 ± 0.08	0.154
WHG	0.58 ± 0.06	0.64 ± 0.11	0.073

Values are presented as mean±SD; MD values measured at the TB, SON, IC, MGB, AR and WHG of the good outcome group of SNHL patients were compared with the poor group.

### Correlation analysis

Only FA values showed statistically significant positive correlations with CAP scores at the trapezoid body (*r* = 0.610, *P* = 0.002), superior olivary nucleus (*r* = 0.615, *P* = 0.001), inferior colliculus (*r* = 0.702, *P* = 0.001), medial geniculate body (*r* = 0.619, *P* = 0.001), auditory radiation (*r* = 0.565, *P* = 0.004) and white matter of Heschl's gyrus (*r* = 0.661, *P* = 0.004). MD values did not displayed statistically significant correlations with CAP scores. CAP scores showed statistically significant negative correlation with age at implant (*r* = -0.563, *P* = 0.004), but no correlationgs of FA and MD values with age at implant at all of the ROIs were observed (*P*>0.05).

## Discussion

In normal hearing subjects, myelinated fibers extend projections to the auditory cortex, from 1 to 12 years-old, and then the development of axon tends to be stable. The formation of auditory cortical myelination is dependent on stimulation by a variety of sounds [9,10]. Emmorey et al. found that auditory deprivation resulted in less myelination and/or fewer fibers projecting to and from auditory cortices, or greater axonal pruning in the congenitally deaf [11]. Although conventional CT/MRI examination is helpful in providing an adequate morphological picture of the auditory pathway [1,12], it cannot evaluate disease processes at the microscopic level, as exemplified in our study where all patients displayed normal brain manifestation based on conventional MRI. Previous studies have shown that FA is a sensitive marker for myelination and microstructural integrity of the brain [13–15]. It has been introduced to assess the structural integrity of CNS and disease conditions that may disturb tissue structural coherence [16,17]. The reduction in FA value in certain regions along the auditory pathway in participants with SNHL had been found, suggesting that there were subtle changes in the integrity of auditory pathway in patients with SNHL [5,7,8]. In this current study, we investigated more regions along the auditory neural pathway (trapezoid body, superior olivary nucleus, inferior colliculus, medial geniculate body, auditory radiation and white matter of Heschl's gyrus) by using DTI to further evaluate the integrity of the auditory neural pathway in SNHL patients. Our findings showed that FA values were decreased, suggesting the presence of a dysmyelinating process in the underlying microstructures along the auditory pathway. On the other hand, MD values remained unchanged, indicating the integrity of axonal fiber tracts. The orientation of the neural fibers was intact [18].

Cochlear implantation is the most effective procedure for the management of severe-to-profound sensorineural hearing loss [19]. While often successful in restoring hearing to the deaf child to some extent, there is still a considerable amount of patients who fail to get a good outcome after the surgery, and furthermore, did not acquire effective speech processing. Therefore, preoperative evaluation in predicting postoperative prognosis in pediatric cochlear implant recipients is important and necessary. Basic neuro-otologic and otolaryngologic evaluations, such as auditory evoked responses (AERs), are often used to assess hearing and localize lesions within the intracranial auditory pathways [20]. However, once the pathway (and therefore the passage of the signal) is disrupted, more distal locations in the pathway are difficult to assess, and for imaging evaluation, to the best of our knowledge, there is only one report that undertook DTI for estimating the surgical outcome of CI, with the findings of significantly higher FA values at the several brain areas, including Broca’s area, genu of the corpus callosum, and auditory tract, in good outcome subjects compared to poor outcome subjects, and in which correlations between FA and CAP scores and open sentence scores are also found [21]. However, a limiting factor was that the DTI scans were performed after surgery, so it was not possible to exclude unknown confounding factors that might affect recovery of the neural integrity in the good/poor outcome group after CI. In our study, we investigated whether there were differences in indices of DTI (FA and MD values) along the central auditory pathway that determined good and poor outcome of CI patients. The main finding of the current study is that there are reductions in FA values and MD values remain unchanged at the trapezoid body, superior olivary nucleus, inferior colliculus, medial geniculate body, auditory radiation and white matter of Heschl's gyrus in poor outcome subjects compared to good outcome subjects. These findings suggested that, compared to poor outcome subjects, good outcome subjects show better myelination and neural integrity along the central auditory pathway, indicating the conservation of microstructural integrity of the central auditory pathway should be an important consideration for CI outcome. In addition, correlation analyses revealed strong correlations between FA values and CAP scores, which suggested that FA values represented valuable predictive biomarkers of CI outcome. On the other hand, CAP scores showed statistically significant negative correlation with age at implant, which provided more support for the perspective that early cochlear implantation might be more effectively to restore hearing in SNHL patients.

It should be noted that there exist some limitations in the current study. Due to the patient population being relatively small, we could not separate the profound SNHL patients according to their severity of hearing loss. However, the widespread distribution of FA values among patients seems to suggest that there might be a correlation between severity of hearing loss and the degree of reduction in FA values, according to a previous study by Chang et al. [5]. The other possible limitation of this study is that, although correlation analysis showed a close correlation between FA values and CAP scores at all of the ROIs investigated along the central auditory pathway, the CAP scores of all the patients were determined 12 months after CI. Therefore, a further study with a larger population of subjects and a longer period of time after CI are essential to confirm the current findings.

In conclusion, the decrease of FA values in locations along the central auditory pathway in profound SNHL patients is most likely a reflection of underlying microstructural changes, and can be attributed to a developmental delay in myelination and/or a loss of axonal fibers. Compared to poor outcome subjects, good outcome subjects after CI show better neural integrity at brain areas associated with auditory functions, and strong positive correlations between the neural integrity and clinical outcomes, statistically significant negative correlation between the clinical outcomes and age at implant are also shown, suggesting that the conservation of microstructural integrity of these brain areas may be important for the outcome of CI and early cochlear implantation might be more effectively to restore hearing in SNHL patients. Preoperative DTI imaging can be used to evaluate microstructural alterations in the auditory neural pathway that are not detectable by conventional MR imaging, and may play an important role in the evaluation of the outcome of surgery.
